# Lysine methylation modifications in tumor immunomodulation and immunotherapy: regulatory mechanisms and perspectives

**DOI:** 10.1186/s40364-024-00621-w

**Published:** 2024-07-30

**Authors:** Yiming Luo, Junli Lu, Zhen Lei, He Zhu, Dean Rao, Tiantian Wang, Chenan Fu, Zhiwei Zhang, Limin Xia, Wenjie Huang

**Affiliations:** 1 Hepatic Surgery Centre, Tongji Hospital, Tongji Medical College, Hubei Key Laboratory of Hepato-Pancreato-Biliary Diseases, Huazhong University of Science and Technology, Wuhan, 430030 Hubei China; 2 Clinical Medicine Research Center for Hepatic Surgery of Hubei Province, Key Laboratory of Organ Transplantation, Ministry of Education and Ministry of Public Health, Wuhan, 430030 Hubei China; 3 Department of Gastroenterology, Institute of Liver and Gastrointestinal Diseases, Hubei Key Laboratory of Hepato-Pancreato-Biliary Diseases, Tongji Hospital of Tongji Medical College, Huazhong University of Science and Technology, Wuhan, 430030 Hubei China

**Keywords:** Lysine methylation, Epigenetic, Cancer immunotherapy, Immunomodulation, Lysine methyltransferases (KMTs), Lysine demethylases (KDMs)

## Abstract

Lysine methylation is a crucial post-translational modification (PTM) that significantly impacts gene expression regulation. This modification not only influences cancer development directly but also has significant implications for the immune system. Lysine methylation modulates immune cell functions and shapes the anti-tumor immune response, highlighting its dual role in both tumor progression and immune regulation. In this review, we provide a comprehensive overview of the intrinsic role of lysine methylation in the activation and function of immune cells, detailing how these modifications affect cellular processes and signaling pathways. We delve into the mechanisms by which lysine methylation contributes to tumor immune evasion, allowing cancer cells to escape immune surveillance and thrive. Furthermore, we discuss the therapeutic potential of targeting lysine methylation in cancer immunotherapy. Emerging strategies, such as immune checkpoint inhibitors (ICIs) and chimeric antigen receptor T-cell (CAR-T) therapy, are being explored for their efficacy in modulating lysine methylation to enhance anti-tumor immune responses. By targeting these modifications, we can potentially improve the effectiveness of existing treatments and develop novel therapeutic approaches to combat cancer more effectively.

## Background

Protein methylation is a major type of post-translational modification (PTM), mainly affecting lysine, arginine, and histidine residues [1, 2]. Lysine methylation stands out as a particularly widespread PTM, intricately regulating histones and non-histone proteins to influence a wide range of physiological or pathological processes [3]. Lysine methylation is a complex biochemical process that occurs when a lysine methyltransferase recognizes a specific lysine residue of a target protein and forms a covalent bond with it. The precision of this process makes it possible for lysine methylation to trigger complex cascade effects on protein molecules that are important for many different cellular functions and biological processes [4].

Numerous studies have shown that protein lysine methylation and demethylation modifications are closely associated with tumor progression [2], and an increasing number of tumor therapies targeting methylation/demethylation have also emerged [5]. Recent research indicates that lysine methylation significantly influences immune regulation within tumors [6–8]. This modification directly impacts the activation and function of immune cells in the tumor microenvironment, thus regulating antitumor immunity. Moreover, it affects tumor recognition and clearance by the immune system through processes such as antigen presentation and T cell infiltration. Our paper reviews the involvement of protein lysine methylation/demethylation in tumor immunomodulation and explores the therapeutic potential of targeting KMTs/KDMs in cancer immunotherapy.

### Lysine methylation

**Fig. 1:**
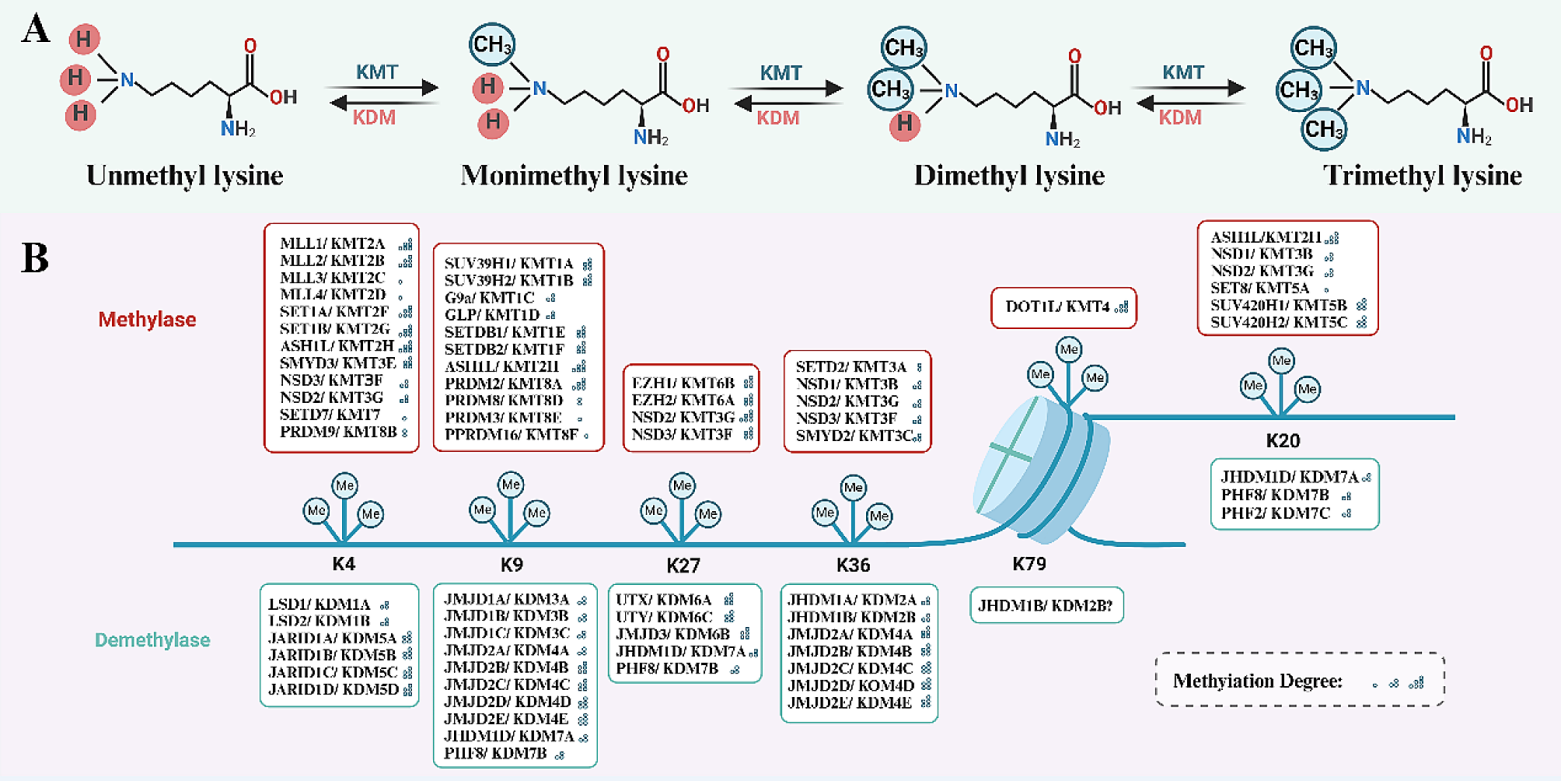
Overview of lysine methylation. (**A**) The processes of lysine methylation and demethylation are depicted. Lysine methyltransferases (KMTs) catalyze the addition of methyl groups onto substrates, while lysine demethylases (KDMs) remove methyl groups, resulting in mono-, di-, and trimethylation of lysine residues. (**B**) Typical lysine methylation sites on core histone proteins H3 and H4 are illustrated

Lysine methylation requires a methyl donor, usually S-adenosylmethionine (SAM). First, SAM is transported with the lysine residue to bind to the catalytic pocket of the SET structural domain. In this process, the ε-amine of lysine is deprotonated by a nearby tyrosine residue for methyl transfer. Next, the lysine chain makes an affinity attack on the methyl group of the SAM so that the methyl group is transferred to the lysine side chain to form the lysine methylation product [9]. The lysine methylation product is then released and the methyltransferase returns to its initial state, ready for the next round of catalysis [10]. The ε-carbon-nitrogen bond of the lysine residue is rotated, which further deprotonates the ε-amine and aligns the resulting lone pair with the methyl-sulfur bond of the SAM for further polymethylation processes. Unlike arginine, which undergoes only mono- and dimethylation modifications, lysine can be methylated to monomethyl lysine (Kme1), dimethyl lysine (Kme2), or trimethyl lysine (Kme3) (Fig. 1A). Different levels of methylation can affect the function of proteins to varying degrees depending on the specific methyltransferase and reaction conditions [11, 12].

### Histone lysine methylation

In recent years, studies based on extensive proteomic mass spectrometry have shown that lysine methylation occurs in thousands of human proteins [13]. Notably, human histones contain many evolutionarily conserved lysine residues, with typical lysine methylation sites located on histone H3 at lysine 4 (H3K4), lysine 9 (H3K9), lysine 27 (H3K27), lysine 36 (H3K36) and lysine 79 (H3K79), as well as on histone H4 at lysine 20 (H4K20) (Fig. 1B) [14].

Histone lysine methylation modifications have important roles in epigenetic regulation and are involved in gene expression, cell differentiation, development, and disease [15]. H3K4 methylation are mainly associated with enhancer and promoter activity and regulate gene expression [16]. Specifically, H3K4me1 mainly marks enhancer regions, especially potentially active enhancers, and promotes enhancer activity through binding to specific transcription factors and other epigenetic marks (e.g., H3K27ac) [17]. H3K4me2 and H3K4me3, on the other hand, are more associated with promoter regions, indicating an open chromatin state, which facilitates the binding of transcription factors and RNA polymerase and thus promote gene expression [18]. These modifications are mainly catalyzed by methyltransferases of the KMT2 family, which includes members such as MLL1/2, while demethylases of the KDM1 and KDM5 families are responsible for removing these methylation marks.

H3K9 methylation are closely associated with gene silencing and heterochromatin formation [19].H3K9me1 is associated with gene silencing and initial alterations in chromatin structure, whereas H3K9me2 and H3K9me3 are found predominantly in the heterochromatin regions of the genome, maintaining gene silencing, and are commonly found in the vicinity of the mitophagy and in non-coding regions [20]. Methylases such as the KMT1 family of methylases are responsible for methylation modifications of H3K9, while the KDM3 family remove these modifications [21].

H3K27 methylation regulate developmental gene expression and cell fate through Polycomb complex-mediated gene silencing [22, 23]. Polycomb repression complex-2 (PRC2)-mediated modification of H3K27me3 can recruit Polycomb repressive Complex 1 (PRC1), which enhances gene silencing by regulating the three-dimensional structure of chromatin through multiple mechanisms (e.g., ubiquitination of H2A) to make it more compact, thereby inhibiting the binding of transcription factors and RNA polymerases [24, 25]. EZH2, a core member of PRC2, is responsible for the methylation of H3K27, whereas the KDM6 and KDM7 families are responsible for demethylation [26].

H3K36 methylation are associated with transcriptional elongation and DNA repair and are mainly concentrated within active gene bodies [27]. H3K36me1 is responsible for labeling of active gene bodies, while H3K36me2 & H3K36me3 are functionally associated with transcriptional elongation, RNA processing and regulation of gene expression [28]. The KMT3 family is responsible for methylation of H3K36me3 and the KDM2 family is responsible for demethylation.

H3K79 methylation are closely associated with gene expression, DNA damage response, and cell cycle regulation [29]. DOT1L is the only known H3K79 methyltransferase. DOT1L enhances the transcriptional activity of genes by regulating chromatin structure, making chromatin more open, and promoting the binding and elongation of transcription factors and RNA polymerase II [30, 31]. At the same time, it recruits DNA damage repair proteins, including 53BP1, to promote the recognition and repair of DNA damage sites [32]. No specific H3K79 demethylases have been identified in the past, although it was recently reported that KDM2B/ JHDM1B may act as a histone demethylase for H3K79me2/3 and link its function to transcriptional repression through sirt1-mediated chromatin silencing [33].

H4K20 methylation have been associated with chromatin structural stability, gene silencing, and DNA repair. H4K20me1 is associated with DNA repair and chromatin structure, marks S-phase chromatin, and is involved in the DNA damage response [34]. H4K20me2 has been associated with gene silencing and genome stability, particularly in heterochromatin regions [35]. H4K20me3 is predominantly found in heterochromatin regions and is associated with strong gene silencing and chromatin structural stability [36]. The KMT5 family is primarily responsible for the methylation of H4K20 and the KDM7 family removes these methylation modifications [37].

Depending on the amino acid position and methylation status of their modification sites, methylation of histones can result in either activation or repression of gene transcription [2]. Typically, H3K9me3, H3K27me3, and H4K20me2/3 facilitate transcriptional repression, while H3K4me1/2/3, H3K9me1, H3K27me1, H3K36me1/2/3, and H3K79me1/2/3 promote transcriptional activation [12]. The dynamic balance of these methylation modifications is critical for normal cellular function, and their dysregulation has been linked to a variety of diseases, including cancer, neurodegenerative diseases, and developmental disorders. Therefore, a deeper understanding of the biological functions and regulatory mechanisms of these modifications is important for the development of new therapeutic strategies.

### Lysine methyltransferases (KMTs)

The structural domains currently considered to have lysine methyltransferase activity in the human proteome can be divided into two main classes. One class is represented by SUV39H1, a methyltransferase with an evolutionarily conserved SET (Su(var)3–9, Zeste enhancer and Trithorax) domain [14]. SUV39H1 was first identified as a KMT in 2000, and was reported to be able to methylate human histone H3K9 as well as being genetically conserved from yeast to humans [38]. Subsequently, many proteins with SET structural domains were successively discovered, approximately 55% of which showed methylation activity towards histones or other proteins [10, 39].

Another large class of methyltransferases are the 7-β-strand (7BS) proteins with typical core folds [40]. After the first 7BS KMT, DOT1L, was identified as a H3K79 lysine methyltransferase in 2002, 16 7BS KMTs have been discovered and characterized to date [10, 41]. Compared with SET structural domain proteins, the substrates of 7BS KMTs mainly include DNA and proteins. Thus, the two classes of KMTs play different functions, whereby the SET protein family is mainly involved in chromatin remodeling, transcriptional regulation, and epigenetic regulation [42], whereas the 7BS family participates in transcriptional regulation, DNA repair, cell cycle control, and embryonic development [43]. In recent years, KMTs have been classified into 12 families based on structural domain characterization and substrate specificity (Table 1).

**Table 1:** Summary of histone methylation sites and structural domains of 12 KMT family members

KMT Family	Member	Histone Methylation Sites	Structural Domain Characterization
KMT1	SUV39H1	H3K9me2/3	SET
	SUV39H2	H3K9me2/3	SET
	G9a(EHMT2)	H3K9me1/2	SET, Ankyrin repeats
	GLP(EHMT1)	H3K9me1/2	SET, Ankyrin repeats
	SETDB1	H3K9me1/2/3	SET, Tudor
KMT2	MLL1(KMT2A)	H3K4me1/2/3	SET, PHD, FYRN, FYRC
	MLL2(KMT2B)	H3K4me1/2/3	SET, PHD, FYRN, FYRC
	MLL3(KMT2C)	H3K4me1	SET, PHD
	MLL4(KMT2D)	H3K4me1	SET, PHD
	SET1A(KMT2F)	H3K4me1/2/3	SET, RRM, WW
	SET1B(KMT2G)	H3K4me1/2/3	SET, RRM, WW
KMT3	SETD1A	H3K4me1/2/3	SET, WW
	SETD1B	H3K4me1/2/3	SET, WW
	SETD7(SET7/9)	H3K4me1	SET
KMT4	NSD1	H3K36me1/2	SET, PWWP
	NSD2(WHSC1)	H3K36me1/2	SET, PWWP
	NSD3	H3K36me1/2	SET, PWWP
KMT5	SUV4-20H1	H4K20me1/2/3	SET
	SUV4-20H2	H4K20me2/3	SET
KMT6	EZH1	H3K27me1/2/3	SET, CXC
	EZH2	H3K27me1/2/3	SET, CXC
KMT7	DOT1L	H3K79me1/2/3	7-β-strand (7BS)
KMT8	SETD8	H4K20me1	SET
KMT9	PRDM2	H3K9me1/2	SET, PR
KMT10	PRDM1	H3K9me1	SET, PR
KMT11	PRDM3	H3K9me1	SET, PR
KMT12	PRDM16	H3K9me1	SET, PR

## Lysine demethylases (KDMs)

Following the discovery of methyltransferases, an enduring debate persisted regarding the existence of demethylating enzymes. The debate was resolved only when LSD1/KDM1A was identified as the first histone lysine demethylase in 2004. In this pioneering study, it was found that LSD1 specifically demethylates histone H3K4 to suppress the transcription of target genes [44]. Subsequent to the identification of LSD1, another subset of demethylases, the KDM Jumonji C (JMJC) family, was revealed [45]. This group of enzymes utilizes the JmjC domain to oxidize lysine residues, thereby effecting demethylation [9]. Similarly, based on structural domain characterization and substrate specificity, KDMs are classified into seven families (Table 2). Histone lysine residues are subjected to strict regulation by KMTs and KDMs to maintain cellular fates and genome stability [42].

**Table 2:** Summary of histone methylation sites and structural domains of seven KDM family members

KDM Family	Member	Histone Methylation Sites	Structural Domain Characterization
KDM1	KDM1A(LSD1)	H3K4me1/2, H3K9me1/2	AOD, SWIRM
	KDM1B(LSD2)	H3K4me1/2	AOD, SWIRM
KDM2	KDM2A(FBXL11)	H3K36me1/2	JmjC, CXXC, F-box
	KDM2B(JHDM1B)	H3K36me1/2	JmjC, CXXC, F-box
KDM3	KDM3A(JMJD1A/JHDM2A)	H3K9me1/2	JmjC, LXXLL, PHD
	KDM3B(JMJD1B/JHDM2B)	H3K9me1/2	JmjC, LXXLL, PHD
	KDM3C(JMJD1C)	H3K9me1/2	JmjC, LXXLL, PHD
KDM4	KDM4A(JMJD2A/JHDM3A)	H3K9me2/3, H3K36me2/3	JmjC, PHD, Tudor
	KDM4B(JMJD2B)	H3K9me2/3, H3K36me2/3	JmjC, PHD, Tudor
	KDM4C(JMJD2C)	H3K9me2/3, H3K36me2/3	JmjC, PHD, Tudor
	KDM4D(JMJD2D)	H3K9me2/3	JmjC
	KDM4E	H3K9me2/3	JmjC
	KDM4F	H3K9me2/3	JmjC
KDM5	KDM5A(JARID1A)	H3K4me2/3	JmjC, ARID, PHD, ZF
	KDM5B(JARID1B)	H3K4me2/3	JmjC, ARID, PHD, ZF
	KDM5C(JARID1C)	H3K4me2/3	JmjC, ARID, PHD, ZF
	KDM5D(JARID1D)	H3K4me2/3	JmjC, ARID, PHD, ZF
KDM6	KDM6A(UTX)	H3K27me2/3	JmjC, TPR
	KDM6B(JMJD3)	H3K27me2/3	JmjC, TPR
KDM7	KDM7A(JHDM1D)	H3K9me1/2, H3K27me1/2	JmjC, PHD
	PHF8(JHDM1F)	H3K9me1/2, H3K27me1, H4K20me1	JmjC, PHD
	PHF2	H3K9me1/2, H4K20me1	JmjC

### Non-histone lysine methylation

In recent years, there has been increasing evidence that lysine methylation is not limited to histones and can also be found in various other proteins [46, 47]. Several proteomic investigations unveiled numerous new methylated proteins and targeted lysine residues [48–50]. Notably, a surprising number of lysine methylases have been identified in processes associated with tumorigenesis and cancer progression. For example, SMYD3 was recently revealed to promote trimethylation of MAP3K2, activating the MAPK pathway and fostering growth signaling in lung, pancreatic, and potentially other cancers [51]. In addition, G9a and GLP have been found to induce lysine methylation on p53 protein residue 373, thereby impairing its activity [52].

Advances in proteomic techniques, especially mass spectrometry, have enhanced our understanding of protein lysine methylation. Increasing numbers of non-histone lysine methylation modifications have been discovered, which together with histone methylation regulate important life activities of cells. Understanding the regulatory mechanisms of lysine methylation in tumors and exploring related therapeutic strategies holds great scientific and clinical importance.

### Lysine methylation in immune cells

As a pivotal epigenetic alteration, lysine methylation extensively regulates tumor cell functions and phenotypes [5]. In recent years, it has become increasingly evident that lysine methylation not only impacts tumor cell growth and metastasis but also exerts a significant influence on various antitumor immune cells within the tumor microenvironment. Immune cells such as T-cells, B-cells, and macrophages play a crucial role in defending against and eliminating abnormal cells. Recent studies have shown that lysine methylation might alter the function and phenotype of immune cells, thereby impacting their ability to identify and eradicate tumor cells. This has profound implications for the efficacy of antitumor immunotherapy.

**Fig. 2:**
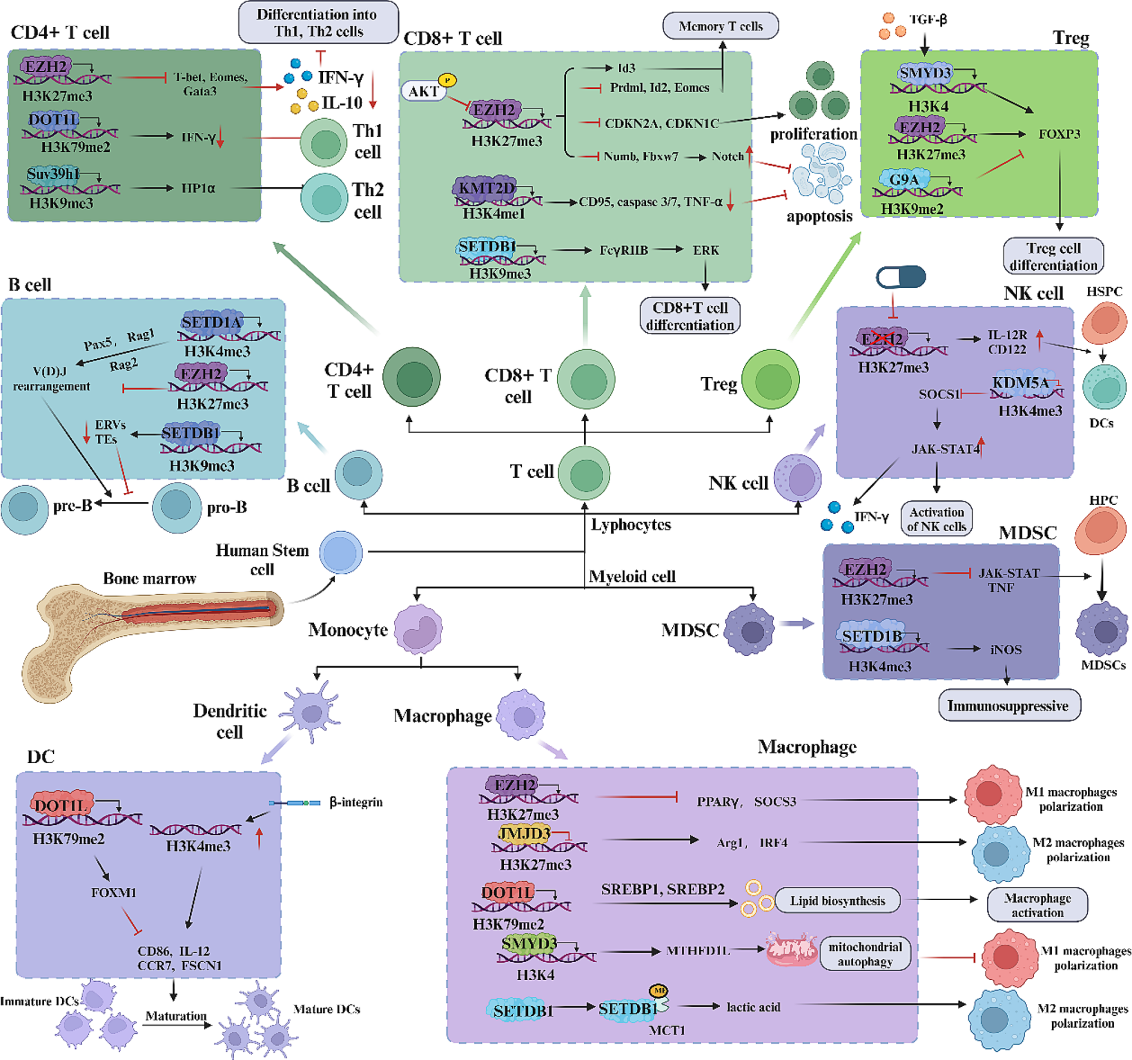
Mechanisms of lysine methylation modifications regulating immune cells. Lysine methyltransferases and demethylases in immune cells modulate important signaling pathways and molecular expression through histone or non-histone methylation, influencing proliferation, differentiation, apoptosis, and activation of lymphoid and myeloid cells

Thus, gaining a comprehensive understanding of the regulatory mechanisms of lysine methylation in these immune cells is imperative for elucidating tumor immune evasion mechanisms and assessing the effectiveness of immunotherapy. This paper aims to summarize recent research advancements on lysine methylation in immune cells (Fig. 2).

### CD8^+^ T cells

CD8^+^ T lymphocytes, pivotal in adaptive immunity, recognize external pathogens and internal cancer cells [53, 54]. When encountering antigenic peptides presented in the context of class I major histocompatibility complex (MHC) molecules, naïve CD8^+^ T cells undergo cellular division, resulting in effector and memory T cells [55, 56]. Investigations of the epigenomic profile of histone modifications in naïve and memory CD8^+^ T cells demonstrated a progressive chromatin remodeling process. H3K27me3 histone modifications are intricately associated with T-cell metabolism, effector function, and the expression of memory-related genes [57]. EZH2 primarily induces gene silencing through catalytic H3K27me3 modification [58–61]. It assumes a critical role in CD8^+^ T memory precursor formation. It has been reported that EZH2 activates Id3 in an H3K27me3-dependent manner while inhibiting Id2, Prdm1, and Eomes. This process facilitates the expansion of memory precursor cells and their differentiation into functional memory cells. Furthermore, Akt activation leads to Ezh2 phosphorylation, attenuating the regulation of related transcriptional programs [62]. Phenotypic analysis of human EZH2^+^ CD8^+^ T cells showed that this subpopulation exhibits enhanced effector capacity and reduced susceptibility to apoptosis [50]. EZH2 inhibits the expression of the cell cycle protein-dependent kinase inhibitors CDKN2A and CDKN1C in activated naïve CD8^+^ T cells via K3K27me3, thus activating the proliferation of CD8^+^ T cells [63]. Furthermore, EZH2 inhibits the Notch inhibitors Numb and Fbxw7 through the same mechanism, thereby activating the Notch pathway and thus stimulating T cells to promote their survival [64, 65]. In addition to EZH2, KMT2D also regulates the survival of activation-induced naïve CD8^+^ T cells by modulating H3K4me1 levels in the enhancer regions of related genes, such as CD95, caspase 3/7 and TNF-α, which are associated with apoptosis and immune function [66]. The Suv39h1-dependent histone H3K9me3 plays a key role in targeting chromatin to silence stem/memory genes during CD8 + T cell differentiation. Suv39h1-deficient CD8 + T cells exhibit sustained survival and increased long-term memory reprogramming capacity [67].

Successful rearrangement of the T cell receptor beta (TCR-β) gene cluster during precursor T-cell maturation is vital for producing double-positive (DP) cells [68]. When the TCR engages self-antigens bearing either class I or class II MHC molecules, immature thymocytes diversify into CD8^+^ or CD4^+^ single-positive (SP) T-cells. In this process, a modest and tightly regulated activation of ERK, crucial for positive selection, is essential [69]. Aberrant expression of the FcγRIIB receptor has been reported in thymocytes that are specifically deficient in the H3K9me3 transferase SETDB1, resulting in the inhibition of the ERK signaling pathway, thereby allowing incremental apoptosis in single CD4^+^ or CD8^+^ thymocytes, which ultimately leads to impaired CD8^+^ T cell development [70].

### T helper (th) cells

CD4^+^ T helper (Th) cells are key immune cells derived from naïve CD4^+^ T cells that protect the body from infections and tumors by coordinating, regulating, and amplifying the immune response [71, 72]. They can differentiate into several subpopulations with different surface molecules, cytokines and key transcription factors expression patterns [73], including Th1, Th2, Th17, and Treg [74]. EZH2 significantly impedes the differentiation of naïve CD4^+^ T cells into Th1 and Th2 phenotypes by promoting H3K27me3 levels of Th-spectrum transcription factors such as T-bet, Eomes, and Gata3, as well as inhibiting the expression of cytokines such as IFN-γ and IL-10 [75, 76]. DOT1L also plays a significant role in constraining Th1 cell differentiation and maintaining lineage integrity. Inhibition of the H3K79me2 activity of DOT1L resulted in a significantly increased abundance of IFN-γ CD4^+^ cells and augmented IFN-γ production [77]. Interestingly, the SUV39H1-H3K9me3-HP1α pathway was recently found to be essential for maintaining Th2 cell stability. Deletion of SUV39H1 alters the H3K9ac to H3K9me3 ratio at the IFN-γ locus, resulting in reduced binding of HP1α at the promoters of silenced TH1 genes [78].

### Regulatory T cells (Tregs)

Specialized CD4^+^ regulatory T cells (Tregs) maintain immune tolerance by suppressing responses [79]. Foxp3 expression is critical for Treg development, and is regulated by both transcriptional and epigenetic mechanisms [80, 81]. Recently, the role of lysine methylation in maintaining the immunosuppressive phenotype of Tregs in cancer has been intensively investigated. Following Treg activation, the FOXP3 binding site shows reduced chromatin accessibility and selective H3K27me3 deposition. This process involves Ezh2 recruitment and downregulation of nearby genes [82, 83]. Treg-specific deletion of EZH2 resulted in reduced levels of H3K27me3, destabilizing FOXP3 expression in activated Tregs. Consequently, these Tregs acquire pro-inflammatory properties, leading to an increase in the number and function of tumor-infiltrating T cells. In mice with colon, prostate and skin cancers, knockdown of EZH2 resulted in a significant inhibition of tumor growth [84, 85]. It has also been reported that methyltransferase SMYD3 directly affects iTreg differentiation by promoting Foxp3 expression through an H3K4-dependent mechanism, and that SMYD3 is transcriptionally regulated by TGF-β1/SMAD3 signaling [86]. Additionally, G9a-mediated H3K9me2 restricts Treg differentiation both in vitro and in vivo by modulating chromatin accessibility and TGF-β1 responsiveness, thereby inhibiting FOXP3 expression [87].

### B cells

B cells act as one of the major antigen-presenting cell types by processing antigenic peptides in the context of MHC molecules, thus mediating the immunogenicity of tumor antigens [88]. H3K4 methylation has been reported to play a crucial role in B cell development [89]. Notably, during the transition from progenitor (pro-B) to precursor (pre-B) B cells, H3K4me3 levels increase in the J gene during IgH locus rearrangement, along with the nearby D gene. This suggests that H3K4me3 is intimately involved in the regulation of V(D)J recombination in the IgH motif during the pre-B phase [90]. For example, the methyltransferase SETD1A controls pro- to pre-B progression by regulating H3K4me3 levels of the B-cell master regulators Pax5, Rag1 and Rag 2 [91]. Similarly, H3K27 methylation plays a pivotal role in B-cell development, with EZH2 controlling IgH rearrangement during early B-cell development in mice [92]. In addition, SETDB1 is constitutively expressed throughout B cell development and is indispensable for its progression [93–95]. In B lymphocytes, Setdb1 aids in the establishment of B-cell-mediated immunity by promoting H3K9me3 to inhibit endogenous retroviruses (ERVs) and transposable elements (TEs), thereby ensuring normal lineage differentiation and ultimately mediating the transition of pro- to pre-B cells [94, 95].

### Natural killer (NK) cells

Natural killer (NK) cells are a subset of cytotoxic lymphocytes that play a key role in immune surveillance against infection and tumors [96, 97]. IFN-γ is one of the markers of NK cell activation [98]. It has been reported that deficiency of H3K4me3 demethylase KDM5A severely inhibits the phosphorylation and nuclear localization of STAT4 while upregulating suppressor of cytokine signaling 1 (SOCS1), leading to the suppression of NK cell activation and reduction of IFN-γ production [99]. Moreover, targeted knockdown of EZH2 in NK cells or the reduction of H3K27me3 levels with small molecule inhibitors notably enhanced the production of IL-15 receptor (IL-15R) CD122^+^ NK progenitors and mature NK cells [100].

### Tumor-associated macrophages (TAMs)

Tumor-associated macrophages (TAMs), a distinct subtype of macrophages found within the TME [101, 102]. While macrophages are traditionally recognized as crucial effectors in immune defense [103], numerous studies have revealed that TAMs possess tumor-promoting characteristics [104].

Different levels of methylation of H3K27 play different roles in the regulation of macrophage polarization and function. The H3K27me3 methyltransferase EZH2 promotes macrophage polarization towards the pro-inflammatory M1phenotype by suppressing the expression of inflammatory molecular pathways such as PPARγ and SOCS3, leading to an increased inflammatory response [105–107]. Conversely, the H3K27 demethylase JMJD3 is able to promote M2-like macrophage polarization by regulating H3K27me3 levels of M2 marker genes such as Arg1, Fizz1, and IRF4 [108–110]. Inhibition of JMJD3 expression disrupts M2 polarization, leading to a pro-inflammatory M1 phenotype [111].

Recently, the influence of cellular metabolism on macrophage function has attracted increasing attention [112], and lysine methylation can affect macrophage polarization by influencing multiple metabolic pathways. SREBP1 and SREBP2 are major transcriptional regulators of fatty acid and cholesterol synthesis, respectively [113]. Notably, blockade of SREBP1 and SREBP2 results in macrophages exhibiting excessive inflammation [114, 115]. Dot1L controls genes related to lipid biosynthesis by inhibiting the H3K79me2-mediated regulation of SREBP1 and SREBP2. Consistently, knockdown of DOT1L in myeloid cells was found to decrease the stability of atherosclerotic plaques and increase the activation of pro-inflammatory plaque macrophages [116]. SMYD3 controls the mitochondrial metabolic enzyme MTHFD3L via H3K4me3 histone methylation, promoting formate synthesis and inducing mitochondrial autophagy, thus hindering M1 macrophage polarization [117]. Lactic acid, a product of the cellular glycolytic process, promotes M2-like macrophage polarization and tumor growth [118]. SETDB1 methylates K473 of the lactate transmembrane transporter protein MCT1, which impedes MCT1-TOLLIP interaction and inhibits TOLLIP-mediated autophagic degradation of MCT1, leading to M2 polarization of TAMs in colorectal cancer [119].

### Myeloid-derived suppressor cells (MDSCs)

Myeloid-derived suppressor cells (MDSCs) constitute a cluster of immature myeloid-derived cells originating from myeloid precursors within the bone marrow [120]. Within the TME, they facilitate T-cell apoptosis and impede antitumor immunity, fostering cancer progression by diminishing the expression of distinct recognition receptors on the surface of T cells [121, 122].

The Jak-STAT and TNF signaling pathways play crucial roles in promoting the proliferation and activation of MDSCs [120, 123, 124]. EZH2 was found to inhibit the differentiation of hematopoietic progenitor cells (HPCs) into MDSCs by inhibiting the Jak-STAT and TNF signaling pathways via H3K27me3 modification [125]. Moreover, the reduction of H3K27me levels in EZH2 using GSK126 was able to increase the generation of MDSCs [126]. In addition, elevated iNOS expression is a hallmark of MDSCs and a key mediator of their immunosuppressive function [127]. SETD1B was reported to promote iNOS expression in tumor-induced MDSCs by increasing H3K4me3 levels [128].

### Dendritic cells (DCs)

Dendritic cells (DCs) serve as instigators of the body’s adaptive immune response, crucially influencing antitumor immunity by internalizing tumor-associated antigens and presenting them to T and B cells [129, 130]. FOXM1 has been reported to inhibit the maturation of bone marrow-derived dendritic cells (BMDCs) by promoting the transcription of Wnt5a in pancreatic and colon cancer. In addition, the methyltransferase DOT1L was found to promote the expression of FOXM1 through H3K79me3, consequently delaying the maturation of DCs in the mouse TME [131]. Additionally, upregulation of β2-Integrin in DCs leads to increased H3K4me3 levels of genes such as CD86, IL-12, CCR7, and FSCN1, thereby promoting DC maturation [132].

### The role of lysine methylation in tumor immune escape

There are a number of complex interactions between tumor cells and components of the immune system. The process begins with tumor cells releasing novel tumor-associated antigens (TAAs), which are detected by antigen-presenting cells such as DCs, B cells, and macrophages, leading to the presentation of antigenic peptides in the context of MHC molecules. Upon recognizing these complexes through the TCR, T cells become activated, leading to the upregulation of CD40L on the surface of Th cells [133]. The interaction of CD40L with CD40 on the surface of DCs further induces the expression of B7, which binds to CD28 on the surface of Th cells, initiating dual signaling that further activates T cells. This activation cascade results in the activation of effector T cells by TAAs. Activated CD8^+^ effector T cells identify the antigenic peptide-MHC-I complex via the TCR, leading to the elimination of targeted cancer cells [134]. However, tumors can evade the immune system and persistently grow by altering the TME [135]. The immune system modulates tumor development by either amplifying or suppressing regulatory signals, a process termed the “cancer-immune cycle” [134, 136].

**Fig. 3:**
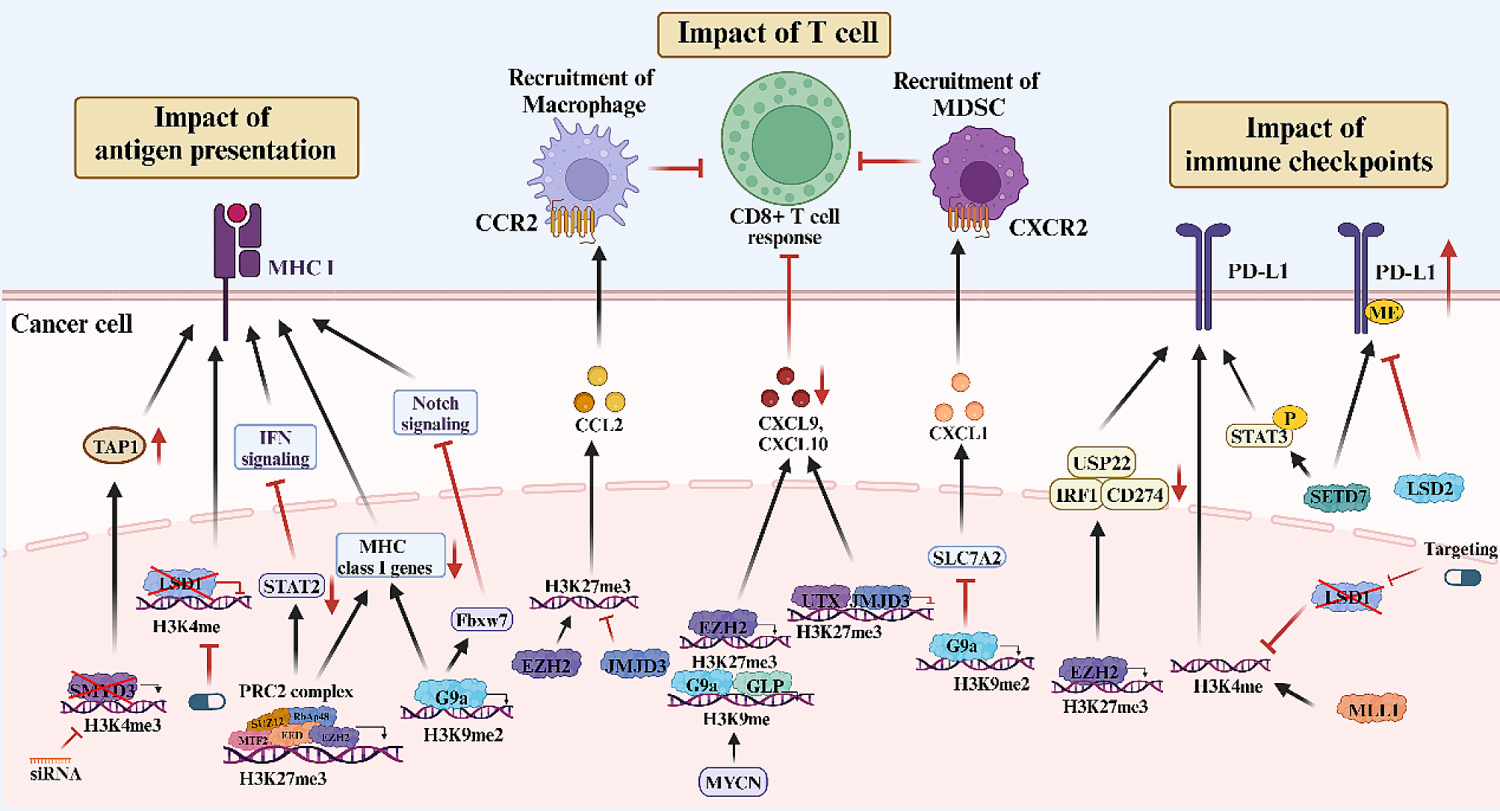
Impact of lysine methylation modifications on immune evasion. Lysine methylation modifications play indispensable roles in regulating immune evasion in various tumors. They affect antigen presentation by influencing MHC class I molecule expression, alter T cell function through modulation of T cell chemoattractant, and influence immune checkpoint regulation

As mentioned above, lysine methylation modifications affect the differentiation, maturation, proliferation, and apoptosis of various immune cell types by modulating their transcriptional regulatory network as well as signal transduction. Furthermore, lysine methylation can alter the immunogenicity and immune evasion capacity of tumor cells, consequently influencing tumor recognition and elimination by the immune system. Here, we delineate the effects of lysine methylation on tumor immune evasion from three perspectives: impact on antigen presentation, impact on T cell immunity, and impact on immune checkpoints (ICs) (Fig. 3).

### Impact on antigen presentation

In order to elicit an effective antitumor response, tumor cells must present neoantigens to the immune system to trigger recognition and killing by CD8^+^ T cells [137]. However, defects in tumor antigen processing and presentation functions, such as deficiency of MHC class I molecules, stands as a primary mechanism through which tumors avoid immune detection and evade eradication by CD8^+^ cytotoxic T cells [138].

A genome-wide CRISPR/Cas9 screen revealed that the PRC2 complex significantly represses mRNA expression of proteins related to the MHC-I antigen-processing pathway by increasing H3K27me3 levels [139]. Similarly, IFN-I expression was found to be silenced by H3K27me3 in breast cancer, while inhibition of EZH2 promoted STAT2-activated IFN signaling and MHC I expression [140]. It was also found that BRD4 could recruit G9a to regulate H3K9 methylation and inhibit the expression of MHC class I genes [141]. In glioblastoma multiforme (GBM), G9a can repress Fbxw7 transcription by promoting H3K9 methylation on the Fbxw7 promoter. Downregulation of Fbxw7 activates the Notch pathway in glioma stem cells, leading to downregulation of MHC I and promotion of PD-L1 expression, thereby suppressing the immune response [142]. An in vitro study demonstrated that SMYD3 knockdown partially inhibited the expression of the antigen-processing protein TAP1 in SCCHN by reducing the level of H3K4me3 [143]. In small-cell lung carcinoma (SCLC), the expression of LSD1 was inversely correlated with the expression of genes related to antigen presentation [144]. Moreover, targeting LSD1 H3K4 demethylase activity restored MHC-I expression and activated the antigen-presenting pathway [144, 145].

### Impact on T cells

In tumor tissues, tumor cells can also modulate T-cell chemokine secretion through lysine methylation to promote or inhibit tumor immune escape, depending on the context. In medulloblastoma, the demethylase UTX/KDM6A can promote the secretion of the th1-type chemokine CXCL9/CXCL10 via H3K27me3 demethylation, thereby facilitating the recruitment of CD8^+^ T cells into the TME [146]. By contrast, EZH2-mediated H3K27me3 suppresses the expression of CXCL9 and CXCL10 in ovarian cancer (OV) cells, thereby hindering the trafficking of effector T cells. Additionally, tumor EZH2 levels exhibited a negative correlation with tumor-infiltrating CD8^+^ T cells and were associated with a poorer patient prognosis [147]. This regulatory mechanism of EZH2 is also present in colon cancer and can be inhibited by the H3K27 demethylase Jmjd3 [148]. In neuroblastoma (NB), MYCN-induced upregulation of the H3K9 methyltransferases G9a and GLP as well as EZH2 can also inhibit IFN-γ-induced expression of CXCL9 and CXCL10 [149]. These studies at least partly explain why many patients seem to benefit little from single-agent immune checkpoint blockade (ICB) therapy.

However, tumor cells can also evolve the ability to recruit immunosuppressive cells, such as TAMs and MDSCs, to the tumor site, forming a suppressive immune microenvironment via chemokine secretion [150]. In this context, the H3K27 methylation level was reported to play an important role in the regulation of macrophage chemokines. In SCLC, the EZH2-mediated H3K27me3 modification of the gene enhancer region inhibits CCL2 expression, resulting in reduced macrophage infiltration and skewed polarization toward the M1 phenotype [151]. Similarly, EZH2 inhibitors abrogated the increase of H3K27me3 levels on the promoter of CCL2 to increase its transcription and secretion in breast cancer, which induced M2 macrophage polarization and recruitment in the TME. This may contribute to the suboptimal efficacy of EZH2 inhibitors in breast cancer treatment [152]. In melanoma, the demethylase JMJD3 promotes macrophage recruitment by decreasing H3K27 methylation levels and transcriptionally upregulating CCL2 [153]. In addition, it has been reported thatg9a-mediated H3K9me2 silences the expression of SLC7A2 in hepatocellular carcinoma (HCC), which results in the upregulation of CXCL1 expression and recruitment of MDSCs [154].

### Impact on immune checkpoints

ICs are a crucial regulatory mechanism in the immune system, maintaining a balanced immune response to prevent runaway inflammation and autoimmune reactions. However, pathogens or tumor cells may exploit immune checkpoints to evade immune attack, thereby leading to disease progression [155]. Based on their roles in T-cell activation, ICs fall into the two categories of co-stimulatory (e.g., CD28, CD80/CD86) and co-inhibitory molecules (e.g., PD-1/PD-L1, CTLA-4) [156]. In the TME, cancer cells can express PD-L1, which inhibits antitumor immune responses by counteracting T-cell activation signals through its interaction with PD-1 on the immune-cell surface [157, 158].

H3K9me3 and H3K27me3 often induce chromatin compaction in promoter regions, potentially suppressing the activation of gene transcription [159, 160]. There was a notable decrease in H3K9me3 and H3K27me3 in the promoter regions of PD-1, CTLA-4, TIM-3, and LAG-3 within the breast cancer TME, potentially resulting in increased expression of these genes [161]. Similarly, H3K9me3 and H3K27me3 play roles in upregulating the CTLA-4, TIGIT, PD-1, and TIM-3 genes in CRC [162, 163]. The H3K27 methyltransferase EZH2 has been reported to inhibit PD-L1 expression in HCC by promoting the H3K27me3 modification in the promoters of CD274, which encodes PD-L1, and the interferon regulatory factor 1 (IRF1) gene [164]. By contrast, the deubiquitinating enzyme USP22 promotes PD-L1 stabilization in colon cancer (COAD), while EZH2 inhibits USP22 transcription via H3K27me3, leading to PD-L1 degradation [165].

The methylation level of H3K4 likewise impacts ICs [166]. The H3K4 demethylase LSD1 plays a crucial role in immune checkpoint regulation within tumor cells. Inhibition of LSD1 was able to promote PD-L1 expression by boosting H3K4me2 at the PD-L1 promoter [167–170]. Alongside LSD1, the H3K4 methyltransferase MLL1 promotes PD-L1 transcription by increasing H3K4me3 levels at the cd274 (PD-L1) promoter in pancreatic cancer cells [166].

Beyond histones, non-histone methylation also significantly influences IC regulation. In non-small cell lung cancer (NSCLC), SETD7 triggers PD-L1 K162 methylation, a process counteracted by LSD2 demethylation. Hypermethylation of PD-L1 at K162 leads to anti-PD-L1 and anti-PD-1 treatment insensitivity, acting as an adverse predictive factor for these treatments in NSCLC patients [171]. In bladder cancer (BCA), SETD7 can also act through a non-histone pathway to directly bind and activate STAT3, leading to increased PD-L1 expression [172].

### Lysine methylation as a molecular target for cancer therapy

#### Lysine methylation inhibitors

There has been a gradual emergence of therapies targeting tumor cell methylation levels for cancer treatment. A number of inhibitors targeting one or more methyltransferases have entered clinical trials or even started to be used in the clinic [173]. The development of H3K27me-specific inhibitors has been an active area of research [140, 174]. In 2020, the FDA approved the EZH2 inhibitor tazemetostat for the treatment of epithelioid sarcoma [175]. Another EZH2 inhibitor, GSK126, is undergoing phase I clinical trials (NCT02082977) for the treatment of lymphoma, solid tumors, and multiple myeloma. Drugs targeting LSD1 also have great potential for the treatment of hematological malignancies. Cyclopropylamine-based LSD1 inhibitors increase histone H3K4 methylation, downregulate the expression of leukemia-associated genes HoxA9 and Meis1, inducing apoptosis and differentiation [176]. ORY-1001 is another potent and selective LSD1 inhibitor that increases H3K4me2 levels in target genes, promotes blast differentiation, and diminishes leukemic stem cell capacity in acute myeloid leukemia (AML). Currently, ORY-1001 is undergoing clinical trials in leukemia and solid tumor patients [177].

It is also worth noting that some KMT inhibitors can be used as adjuvants, offering better efficacy when combined with other drugs [177]. In HCC, the combination of the LSD1 inhibitor ZY0511 with DTP3, an inhibitor of the apoptosis-related gene GADD45B, has demonstrated promising results. This combination promoted apoptosis in HCC and effectively inhibited cellular proliferation both in vitro and in vivo [178]. Additionally, several other KMT inhibitors are currently undergoing preclinical studies, underscoring the broad potential of targeting the lysine methylation pathway for treating various cancers.

### Lysine methylation and immunotherapy

Tumor immunotherapy is an emerging paradigm in cancer treatment, harnessing the inherent immune system of the host to counteract neoplastic cells. Diverging from conventional therapeutic modalities such as chemo- and radiotherapy, which principally aim at direct eradication of malignant cells, immunotherapy operates by priming or augmenting the immune milieu to enhance its proficiency in discerning, assaulting, and eliminating cancerous entities, thus establishing control over cancer progression and preventing recurrence [179]. Immunotherapy encompasses diverse methods such as immune checkpoint inhibitors, chimeric antigen receptor T-cell (CAR-T) therapy, and other therapeutic strategies [88]. In recent years, immunotherapy has emerged as a prominent approach in cancer treatment. However, it is important to recognize that while a minority of patients greatly benefits from these treatments, many others develop innate or acquired drug tolerance, ultimately leading to immunotherapy failure [180]. Consequently, there is a pressing need for continued research into combination approaches based on immunotherapy to increase the overall survival rates of patients with advanced cancer.

#### Lysine methylation and immune checkpoint inhibitor (ICI) therapy

Research on immune checkpoints has garnered significant attention in the field of cancer immunotherapy. A crucial strategy in cancer treatment involves bolstering the immune response to tumors by inhibiting these checkpoints, termed immune checkpoint inhibitor (ICI) therapy [181, 182]. This therapeutic approach is currently mostly implemented using antibodies targeting key immune checkpoints, such as PD-1, PD-L1, and CTLA-4. These antibodies function by impeding the inhibitory interaction between cancer cells and immune cells to reactivate the immune response [155, 183].

ICI therapy has demonstrated notable efficacy across diverse cancer types, including melanoma, non-small cell lung cancer, and renal cell carcinoma [184–186]. Despite its success, ICI therapy still has significant limitations, with only a fraction of patients (20–40%) benefiting from it. A primary challenge lies in the low patient response rate, underscoring the necessity for further research to refine treatment protocols [187, 188]. Epigenetic modulation of the tumor microenvironment, particularly lysine methylation modification, enhances the effectiveness of immunotherapy. Here, we summarize recent studies targeting lysine methylation in combination with immune checkpoint therapy (Table 3).

**Table 3:** Combined application of KMTi/KDMi and ICIs in study

Targets	Inhibitor	Combined ICIs	Cancer Types	Effects	References
EZH2	DZNep	PD-L1 antibody	OV	Increases tumor-infiltrating CD8 + T cells and Th1 chemokines	[189]
	EPZ	PD-1 antibody	PCa	Activates a dsRNA–STING–interferon stress axis	[190]
	GSK126	PD-1 antibody	HNSCC	Enhances antigen presentation on tumor cells. increases CD8 + T cell proliferation, IFNγ production and tumor cytotoxicity	[191]
	Taz (EPZ-6438)	PD-1 antibody	COAD	Transcriptionally upregulates USP22 expression, which further stabilizes PD-L1	[165]
	CPI-1205	CTLA-4 antibody	BLCA	Inhibits the phenotype and function of Tregs, enhances the cytotoxic activity of Teffs	[192]
	GSK503	CTLA-4 antibody	Melanoma	Promotes tumor immunogenicity and antigen presentation	[193]
Suv39h1	ETP-69	PD-1 antibody	Melanoma	Inhibits lymphocyte exhaustion and increases their effector capacity	[194]
G9a	UNC0642	PD-1 antibody	Melanoma	Increases LC3B signaling to regulate autophagy, regulates IFN signaling	[195]
MLL1	Verticillin A	PD-1/PD-L1 antibody	PAAD	Inhibits the expression of PD-L1, promotes the effects of FasL and CTL	[196]
LSD1	HCI-2509	PD-1 antibody	TNBC	Promotes PD-L1 expression and increases CD8 + T cell infiltration	[169]
	SP-2509	PD-1 antibody	HNSCC	Decreases Ki-67 levels and increases CD8 + T cell infiltration in the TME	[197]
	SP-2509	PD-1 /PD-L1 antibody	OSCC	Promotes PD-L1 expression and increases CD8 + T cell infiltration	[198]
	ORY-1001	PD-1 antibody	NSCLC	Promotes ERGIC1 transcription, leading to stabilization of IFNGR1 and activation of IFN-γ signaling	[145]
	ORY-1001	PD-L1 antibody	SCLC	Restores antigen presentation and activates intrinsic immunogenicity	[144]
	Bomedemstat	PD-1 antibody	SCLC	Increases MHC-I presentation and T-cell-mediated killing	[199]

In recent years, there has been a steady increase in the number of studies targeting H3K27me3 in combination with immunotherapy. Several animal studies have also shown that targeting EZH2 in combination with anti-PD-1/L1 antibody therapy can be effective in treating various tumors [165, 189–191]. Suv39h1 can inhibit TCR activation, terminal differentiation and ISG expression programs by controlling the levels of H3K9me3 in some stem cell/memory-related genes. The inhibitor ETP-69 showed promise either alone or when paired with anti-PD-1 therapy to bolster antitumor immune responses for melanoma treatment by targeting H3K9me3. When Suv39h1 is inhibited, anti-PD-1 treatment inhibits the lymphocyte exhaustion program and increases effector cell capacity [194]. Additionally, the use of the G9a inhibitor UNC0642 also was found to enhance the effects of immunotherapy in melanoma, and decreasing H3K9 methylation in the promoter region not only increased signaling at the level of LC3B to regulate autophagy, but also modulate IFN signaling, amplifying the impact of anti-PD-1 therapy [195]. In pancreatic tumor cells, H3K4me3 is enriched in the cd274 promoter. Verticillin A-mediated inhibition of MLL1 reduced H3K4me3 levels in the CD274 promoter and PD-L1 expression in tumor cells, coupled with anti-PD-1/PD-L1 antibody immunotherapy, effectively curtailed pancreatic tumor growth [196].

The immunogenicity of tumor cells plays an important role in the T cell-mediated immune response, and the low immunogenicity of some tumors is an important reason for their insensitivity to ICI therapy [200, 201]. In response to this, an immunotherapeutic idea has been developed to improve the effect of immunotherapy by increasing the immunogenicity of tumor cells. One study reported that inhibition of LSD1 in B16 melanoma cells was able to increase H3K4me2 levels, leading to dsRNA stress response activation, which triggered an increase in immunogenicity and T-cell infiltration, sensitizing the tumor cells to anti-PD-1 antibody treatment [167]. Similarly, inhibition of LSD1 in triple-negative breast cancer(TNBC) by RNAi or HCI-2509 promoted the expression of PD-L1 by increasing its level of H3K4me2, resulting in a significant increase of TNBC immunogenicity, significantly inhibiting tumor growth and metastasis in combination with an anti-PD-1 antibody [169]. Similar synergistic effects have also been demonstrated in head and neck squamous cell carcinoma (HNSCC) and oral Squamous Cell Carcinoma (OSCC) [197, 198].

As mentioned previously, LSD1 plays an inhibitory role in tumor antigen presentation. Accordingly, several studies have reported that targeting LSD1 to increase antigen presentation by tumor cells can increase the efficacy of ICB therapy. It was reported that the LSD1 inhibitor ORY-1001 was able to promote ERGIC1 transcription by increasing H3K4me2 levels, leading to IFNGR1 stabilization and activation of IFN-γ signaling, resulting in increased MHC class I expression [145]. Similar findings were validated in SCLC, where inhibition of LSD1 using ORY-1001 and bomedemstat both increased MHC-I expression to promote the effects of anti-PD-1 and PD-L1 antibody therapy, respectively [144, 199]. Furthermore, it has also been proposed that LSD1 inhibition induces TGFβ expression in tumor cells, leading to suppressed cytotoxicity of CD8^+^ T cells, thus limiting the immunotherapeutic efficacy of LSD1 inhibitors. Therefore, the use of triple therapy combining LSD1 inhibition with blockade of TGFβ and PD-1 may provide a new therapeutic strategy for tumors with low immunogenicity [168].

In addition to its effect on PD-1/PD-L1 targeted therapy, lysine methylation modification similarly enhances the effectiveness of other ICI treatments. CTLA-4, a membrane-bound protein expressed on the surface of activated T-cells, produces signals that suppress T-cell immune responses upon binding with its ligands B7-1 (CD80) and B7-2 (CD86). This interaction results in decreased abundance of activated T-cell and impedes memory T-cell formation [202]. Blocking CTLA-4 can boost the body’s immune response against tumor cells, restoring T-cell activity and extending memory T-cell survival [203]. Therefore, drugs targeting CTLA-4 hold significant promise for tumor immunotherapy. The FDA has approved the CTLA-4 inhibitor ipilimumab for adjuvant therapy in stage III melanoma and advanced melanoma [204–206].

Goswami et al. noted an increase in EZH2 expression in peripheral blood T cells of ipilimumab-treated patients. They suggested that inhibiting H3K27me3 in T cells using CPI-1205 could enhance the efficacy of anti-CTLA-4 therapy in bladder cancer and melanoma [192]. Zingg et al. discovered that H3K27me3 upregulation in melanoma resulted in transcriptional silencing of genes associated with immunogenicity and antigen presentation, which can synergistically inhibit melanoma growth through EZH2 inhibition using GSK503, alongside anti-CTLA-4 and IL-2 therapy. The underlying therapeutic mechanism is dependent on IFN-γ production and downregulation of PD-L1 in melanoma cells [193].

In recent years, a number of inhibitors targeting lysine methylation modifications have stepped into preclinical studies and achieved positive efficacy in combination with ICI therapy (Table 4). Notably, tazemetostat, targeting H3K27me3, is undergoing evaluation in a single-arm, open-label phase Ib/II trial in combination with the PD-1 blocker pembrolizumab for advanced non-small cell lung cancer (NCT05467748). Another small molecule inhibitor of EZH2, XNW5004, is also under evaluation in a phase I/II trial in combination with a PD-1 monoclonal antibody for treating advanced solid tumors (NCT06022757). There is also a phase I/II trial evaluating the efficacy of the LSD1 inhibitor bomedemstat in combination with the anti-PD-L1 drug atezolizumab in patients with extensive-stage small-cell lung cancer (ES-SCLC) (NCT05191797). A multicenter, open-label phase I/II study gave a positive assessment of CPI-1205 combined with ipilimumab for advanced solid tumors (NCT03525795).

**Table 4:** Clinical trials of ICIs based on lysine methylation modification inhibitor

Trial identifier	Drug	Targets	Combined ICIs	Cancer Types	Year	Phase	Status
NCT05467748	Tazemetostat	EZH2	PD-1 antibody Pembrolizumab	NSCLC	2024	I/II	Not yet recruiting
NCT06022757	XNW5004	EZH2	PD-1 antibody pembrolizumab	Advanced solid tumors	2023	I/II	Recruiting
NCT03525795	CPI-1205	EZH2	CTLA-4 antibody Ipilimumab	Advanced solid tumors	2019	I	Completed
NCT04407741	SHR2554	EZH2	PD-L1/TGF-β antibody SHR1701	Solid tumors and B-cell lymphoma	2020	I/II	Recruiting
NCT05191797	Bomedemstat	LSD1	PD-L1 antibody Atezolizumab	ES-SCLC	2022	I/II	Not recruiting
NCT04350463	CC-90,011	LSD1	PD-1 antibody Nivolumab	SCLC and NSCLC	2020	II	Completed
NCT04611139	SP-2577	LSD1	PD-1 antibody Pembrolizumab	SCCOHT, OCCC, EOC and EEC	2021	I	Withdrawn

Efforts to combine lysine methylation inhibitors with ICIs are emerging as a groundbreaking approach in tumor immunotherapy. A large number of clinical trials of immune-combination therapies targeting lysine methylation modifications are currently underway, which offers new directions and hope for improving the response rate of immunotherapy.

### Lysine methylation and CAR-T therapy

When a cancer is poorly immunogenic, relying solely on immune checkpoint therapy may not yield desired outcomes [207]. However, new advanced cellular therapies hold the promise to reintroduce a response to immunotherapy in poorly immunogenic cancers [208]. CAR-T cell infusion therapy is currently the most widely investigated modality of cellular therapy. This approach involves integrating a synthetic CAR into the patient’s own T-cells, empowering them to engage tumor cell surface antigens through antigen-binding structural domains, typically single-chain variable fragments (scFvs). The resulting engineered CAR-T cells can eliminate tumor cells devoid of MHC restriction [209]. CAR-T cells primarily execute tumor cell elimination via the granzyme perforin pathway, with the Fas/FasL pathway also playing a significant role in their cytotoxicity against tumor cells [210].

**Fig. 4:**
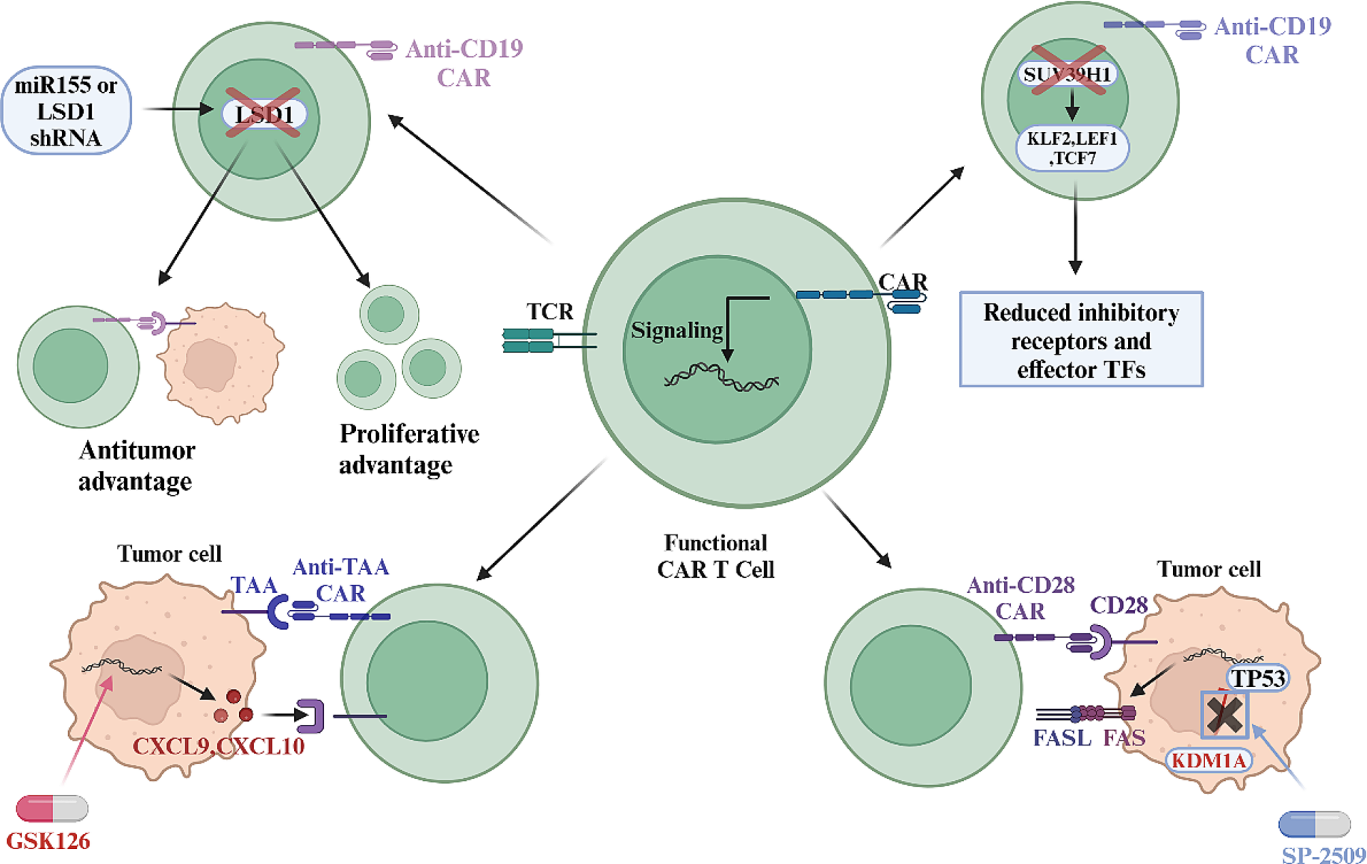
Effects of lysine methylation modifications on CAR-T cell therapy. Enhancing the proliferation capacity and anti-tumor activity of CAR-T cells through genetic editing or targeting the lysine methylation levels of tumor cells with small molecule drugs both contribute to improving the efficacy of CAR-T cell therapy

CAR-T therapies have demonstrated impressive effectiveness in hematological tumors such as B-cell acute lymphoblastic leukemia (ALL) and relapsed/refractory large B-cell lymphoma (LBCL), with cure rates as high as 90% [211]. However, the high incidence of drug resistance persists as a major obstacle for CAR-T cell therapy in solid tumors. Therefore, we need to continue the exploration of CAR-T therapies to find new targets with improved therapeutic potential. As mentioned above, lysine methylation modifications are closely related to T cell function. Studying the role of lysine methylation in CAR-T cell therapy and developing relevant epigenetic strategies are therefore important research directions we cannot ignore. Here, we summarize studies in recent years targeting lysine methylation to increase CAR-T cell therapy (Fig. 4).

Peng et al. constructed TAA-specific CD8^+^ T cells and performed overdose T-cell therapy on an OV model established in NSG mice. They found that GSK126 further enhanced the effectiveness of T-cell therapy. This enhancement is due to the decrease of H3K27me3 levels of Th-1 chemokine CXCL9 and CXCL10 promoter in TME after combination therapy, resulting in increased expression levels and resulting in increased CD8 + t cell infiltration [189]. For neuroblastoma, Sulejmani et al. engineered CAR-T cells targeting the glycosylated CE7 epitope of L1CAM (CD171) [212]. The results revealed that the LSD1 inhibitor SP-2509 sensitized neoantigen-expressing tumor cells to CAR-T cell therapy by releasing an antigen-independent killing signal through the FAS-FASL axis by inhibiting the H3K9me3 level of FAS [213]. It has also been shown that decrease of H3K9me3 living level caused by LSD1 specific knockdown in CD19 CAR T cells will increase the secretion of IFN-γ, TNF-α and IL-2, and improve the killing function of T cells. LSD1-KD CD19 CAR-T cells also secreted more IFN-γ and expanded better in animal models [214]. Recently, it was reported that suppression of H3K9 methylation mediated by specific knockdown of SUV39H1 enhanced CAR-T cell expansion and persistence, improving their antitumor capacity in human leukemia and prostate cancer models [215–217]. In addition to this, it has been reported that CAR-T cell depletion is associated with DNA methylation of genes regulating T cell pluripotency and that CAR-T cell therapies targeting DNMT3A can help to resist CAR-T cell exhaustion.The DNMT3A KO CAR-T cells retained a stem cell-like epigenetic program during prolonged stimulation, which was coupled with the up-regulation of IL-10. coupled to the upregulation of IL-10 [218].

Translated with www.DeepL.com/Translator (free version).

Lysine methylation can improve the duration of therapeutic effects and patient survival by regulating the function and phenotype of CAR-T cells, offering new opportunities for the application of CAR-T cells in the clinic.

#### Lysine methylation and other targeted therapies

In addition to ICIs and CAR-T therapy, the efficacy of tumor immunotherapy can be enhanced by targeting the lysine methylation levels of a variety of immunomodulatory cells [219]. We have also summarized other immunotherapies here (Table 5).

**Table 5:** Lysine methylation and other targeted immunotherapies

Targets	Lysine Methylation Site	Disease	Drug	Mechanism	References
Treg cells	H3K27me3	pan-cancer	EZH2 inhibitor CPI-1205	Promotes pro-inflammatory traits in Treg cells within TME, suppressing immune tolerance	[84, 192]
NK cells	H3K27me3	HCC	EZH2 inhibitor GSK343	Enhances NK cell-mediated clearance of HCC by upregulating NKG2D ligands	[220]
TAMs	H3K79me3	Various Solid Tumors	DOT1L inhibitor EPZ5676	Efficiently induces regression in HCC by coupling with macrophage depletion or NF-κB inhibition	[221]
Antigen presentation	Ku70 Lysine	HCC and CRC	SMYD2 inhibitor AZ505	Activates cGAS-STING pathway by inhibiting Ku70 methylation, leading to sustained DNA damage and incorrect repair, stimulating antitumor immunity	[222]
Antigen presentation	H3K27me3	Melanoma	EZH2 inhibitor GSK126 + STING agonists	Restrains tumor growth and boosts CD8 + T cell infiltration by elevating MHC class I expression and chemokine production	[223]
Antigen presentation	H3K27me3	HNSCC	EZH2 inhibitors GSK126 + EPZ6438	Enhances antigen presentation and promotes antitumor immunity by reducing H3K27me3 modification on the β-2 microglobulin promoter	[191]
SASP	H3K27me3	PDAC	EZH2 inhibitor + Senescence-inducing therapy	Stimulates the production of the SASP chemokines CCL2 and CXCL9/10, leading to enhanced NK and T cell infiltration and PDAC eradication	[224]

There is increasing evidence that Tregs are pivotal in fostering immune tolerance towards tumor cells, representing a barrier to immunotherapy [225, 226]. Tregs induce a state of CTL dysfunction in TME, characterized by reduced expression of T cell effector molecules, reduced release of cytotoxic particles, as well as elevated expression of the co-inhibitory checkpoints PD-1 and TIM-3 [227]. As described above, H3K27me3 level is closely related to Treg function, and employing the EZH2 inhibitor CPI-1205 prompted Treg cells to adopt pro-inflammatory traits within the TME while suppressing immune tolerance [84, 192]. NK cells are primarily responsible for orchestrating innate immune responses against both pathogens and tumor cells. The NKG2D receptor is important for the antitumor immune role of NK cells [228, 229]. Bugide et al. found that HCC, which exhibits reduced expression of NKG2D ligand due to EZH2-mediated transcriptional repression caused by H3K27me3, resists NK cell-mediated clearance. The EZH2 inhibitor GSK343 enhanced the eradication of HCC by NK cells by upregulating NKG2D ligands [220]. TAMs are associated with a dismal prognosis across various solid tumors and dampen the therapeutic effectiveness of ICI therapies [230]. Given the important function that lysine methylation modifications play in the recruitment as well as activation of TAM, a series of immunotherapies targeting lysine methylation levels of TAM have also been developed. A treatment approach coupling the DOT1L inhibitor EPZ5676 with either macrophage depletion or NF-κB inhibition efficiently induced regression in HCC [221].

In addition to targeted cellular therapy, lysine methylation modification therapy can also modulate other steps of the “tumor-immune cycle” to improve the efficacy of immunotherapy. The STING signaling pathway is pivotal for proficient innate immune signaling [231], fostering the production of IFN and numerous other pro-inflammatory cytokines [232, 233]. The SMYD2 inhibitor AZ505 causes sustained DNA damage and incorrect repair by inhibiting Ku70 methylation at lysine-74, lysine-516, and lysine-539 sites, which leads to the accumulation of dsDNA and activation of the cGAS-STING pathway, thereby stimulating antitumor immunity through infiltration and activation of CD8^+^ T cells [222]. In melanoma, H3K27me3 expression was negatively correlated with STING expression at the protein level and combining the EZH2 inhibitor GSK126 with STING agonists elevated MHC class I expression and chemokine production, restraining tumor growth and boosting CD8^+^ T cell infiltration [223]. Targeting EZH2 with GSK126 and EPZ6438 reduces histone H3K27me3 modification on the beta-2 microglobulin promoter and also enhanced antigen presentation and promoted antitumor immunity in head and neck cancer [191]. Inhibition of H3K27me3 levels also stimulated the production of the SASP chemokines CCL2 and CXCL9/10, leading to enhanced NK and T cell infiltration and pancreatic cancer (PDAC) eradication in a mouse model. Combining EZH2 inhibition with senescence-inducing therapy holds promise for achieving immune-mediated tumor control in PDAC [224].

#### Conclusion and prospects

Recently, researchers increasingly focused on the functions and biological effects of lysine methylation. Numerous studies have established the critical role of lysine methylation in regulating diverse physiological processes, including protein structure and function, gene expression, and cellular activities. In addition, dysregulation of lysine methylation was found to be closely associated with tumorigenesis [234, 235]. Moreover, lysine methylation can affect tumor progression by modulating immune activities, which makes it a focus of current research on antitumor immunotherapy [50, 236, 237]. In this paper, we reviewed the significant immunomodulatory effects of lysine methylation within the TME, encompassing the regulation of immune cell behavior, immune evasion mechanisms of tumor cells, and the potential for targeting lysine methylation to improve the efficacy of immunotherapy. Many small molecule inhibitors targeting KMTs/KDMs showed anticancer effects in recent studies. However, we cannot simply infer therapeutic effects only by considering the mechanism against cultured tumor cells, as clinical translation necessitates a broader view of the effects they exert in the TME.

Several strategies have emerged in recent years to improve lysine methylation modification-based cancer immunotherapy, such as combining certain lysine methylation modulators with one or more classical therapeutic regimens (ICIs, classical anticancer drugs, other immunostimulants), or developing CAR-T cell therapies that take advantage of lysine methylation modifications, among others. Combination therapy regimens can lead to better efficacy of immunotherapy and prevent the common phenomenon of acquired resistance to single-agent immunotherapy. Some combination therapy regimens are already showing some promise in early clinical studies.

It has to be acknowledged that the mechanisms by which lysine methylation modifications affect immune and tumor cells are complex and not fully understood. For example, in many studies, EZH2 has been reported to promote the malignant behavior of tumor cells, as well as having significance for Treg development. Consequently, EZH2 ablation or pharmacological inhibition is often deemed effective in curbing tumor growth and enhancing immune activity within the TME. However, He et al. reported that EZH2 also governs the formation of CD8^+^ T memory precursors and their antitumor activity. Additionally, inhibiting EZH2 can compromise TME immunoreactivity [62]. Therefore, targeting different cells may yield varying or even opposite therapeutic effects. This makes it necessary to focus more on the holistic nature of the TME when treating patients with inhibitors. A comprehensive understanding of the precise mechanisms underlying the roles of protein methylation in tumor immunomodulation is indispensable for guiding the development of novel immunotherapeutic strategies.

Future investigations should focus on the heterogeneity of protein methylation across various tumor types to develop more precise therapeutic interventions. In summary, a comprehensive understanding of the mechanistic roles of lysine methylation in tumor immunomodulation can pave the way for the development of innovative immunotherapeutic approaches and strategies to improve the clinical treatment and prognosis of cancer patients.

## Data Availability

No datasets were generated or analysed during the current study.

## Abbreviations

PTM: Post-translational modification
KMTs: Lysine methyltransferases
KDMs: Lysine demethylases
SAM: S-adenosylmethionine
Kme1: Monomethyl lysine
Kme2: Dimethyl lysine
Kme3: Trimethyl lysine
SET: Su(var)3–9, Zeste enhancer and Trithorax
7BS: 7-β-strand
JMJC: Jumonji C
TME: Tumor microenvironment
Th: T helper
Tregs: Regulatory T cells
MHC: Major histocompatibility complex
CTLs: Cytotoxic T lymphocytes
TCR-β: T cell receptor beta
ERVs: Endogenous retroviruses
TEs: Transposable elements
pro-B: Progenitor B cells
pre-B: Precursor B cells
NK: Natural killer
TAMs: Tumor-associated macrophages
APCs: Antigen-presenting cells
MDSCs: Myeloid-derived suppressor cells
DCs: Dendritic cells
BMDCs: Bone marrow-derived dendritic cells
TAAs: Tumor-associated antigens
ICs: Immune checkpoints
GBM: Glioblastoma multiforme
SCLC: Small-cell lung carcinoma
NB: Neuroblastoma
ICB: Immune checkpoint blockade
NSCLC: Non-small cell lung cancer
AML: Acute myeloid leukemia
BCA: Bladder cancer
CAR-T: Chimeric antigen receptor T-cell
ICI: Immune checkpoint inhibitor
TNBC: Triple-negative breast cancer
HNSCC: Head and neck squamous cell carcinoma
OSCC: Oral Squamous Cell Carcinoma
ES-SCLC: Extensive-stage small-cell lung cancer
scFvs: Single-chain variable fragments
ALL: Acute lymphoblastic leukemia
LBCL: Relapsed/refractory large B-cell lymphoma
COAD: Colon cancer
PCa: Prostatic cancer
OV: Ovarian cancer
PDAC: Pancreatic cancer
